# Perceptions and Experiences of Overweight among Women in the Ga East District, Ghana

**DOI:** 10.3389/fnut.2016.00013

**Published:** 2016-06-02

**Authors:** Richmond Nii Okai Aryeetey

**Affiliations:** ^1^Public Health Nutrition Group, Population, Family, and Reproductive Health Department, University of Ghana School of Public Health, Legon, Accra, Ghana

**Keywords:** belief, perception, overweight, experience, women, stigma, Ghana

## Abstract

**Introduction:**

Overweight and obesity are a growing public health challenge among women of reproductive age. While cultural norms suggest preference for an overweight body image, limited evidence exists regarding women’s beliefs and experiences of overweight in Ghana. The current study explored beliefs, perceptions, experiences, and practices concerning overweight among women living in suburban Accra, Ghana.

**Methods:**

Four focus group discussions, and 10 in-depth interviews (IDI) were implemented among 42 adult women (>18 years) seeking preventive child health services in Dome, Accra. All the women in the IDI were overweight. In addition to notes, interviews and discussions were audio-recorded and transcribed for systematic content and narrative analysis.

**Results:**

Overweight was considered undesirable by most women. Overweight individuals were often stigmatized using uncomplimentary names, such as cargo, obolo, and so on. However, some weight gain was admired and expected by women and their family and friends. Weight gain that was considered beautiful was believed to “evolve naturally.” Weight gain that is either medically induced perceived as excessive was not viewed positively. Weight gain by women was perceived as a sign of financial prosperity and good care by a spouse. Overweight was perceived to be linked with heredity, childbirth, gluttony, and contraception. Adverse experiences of overweight included poor self-image, declining social lifestyle, increased disease risk, and feeling tired always. Strategies that had been used in order to lose weight included skipping meals, avoiding carbohydrate-based foods, and drinking herbal teas.

**Conclusion:**

There is admiration for some weight gain among women but when it is excessive, overweight is stigmatized. Misperceptions regarding partner expectations, determinants of overweight, and weight reduction strategies require effective behavior change interventions in Ghana.

## Introduction

An adult is considered overweight if his or her body mass index (BMI) exceeds 25 kg/m^2^. Globally, overweight has become a major public health challenge. An estimated 35% of adults 20 years and above were overweight at the end of 2008 (1). It is estimated that more than half a billion adults are obese, that is, having a BMI in excess of 30 kg/m^2^. There is evidence which further suggests that overweight is increasing at a faster pace in developing countries (2, 3). In most settings, the evolution of overweight and obesity often shows a pattern where women initially have higher rates than men (1).

Overweight is a concern because it is has been demonstrated to have significant health and social impacts. The risk of cardiovascular diseases, diabetes mellitus, and mortality due to these conditions are known to increase with increasing BMI (4–7). Furthermore, among women in reproductive age, obesity is associated with increased risk of delivery complications as well as increased risk of non-communicable disease in their offspring (8). In developing countries where capacity to manage overweight and related non-communicable disease is limited, a double burden of overweight and undernutrition, together with a high burden of communicable diseases presents a particularly challenging public health scenario (9). In addition, excessive overweight is also known to elicit stigma and prejudice, leading to adverse social and economic outcomes, including low self-esteem, depression, and poor school achievement and employment prospects as well as suboptimal productivity (5, 10–12). Studies among different population groups suggest that perception of body size influences decisions about weight gain (13, 14). In some cultures, body weight and size may be linked with cultural norms and expectations of appearance as well as risk behaviors (15).

In Ghana, recent Demographic and Health Surveys have estimated that between 30 and 40% of women in reproductive age are overweight (16, 17). In urban areas, estimates of overweight exceeds 50% of women. This represents an increase of more than 70% from the prevalence observed earlier in the 2003 DHS (18). Globally, significant changes in food system components and their accessibility, as well as the resultant changes in lifestyle have been implicated as part of the explanation for the observed trend (2).

Thus, public health interventions to address overweight have often been designed to elicit changes in dietary and physical activity behavior. Little is known, however, about individual beliefs and practices regarding overweight and obesity and how these can be incorporated into control strategies in the Ghanaian setting. It is known that community and individual perceptions can influence the choices about diets and physical inactivity (19), which are the key direct determinants of overweight. The current study was designed to describe the beliefs, perceptions, and experiences of urban-dwelling Ghanaian women regarding overweight. Understanding the beliefs and perceptions of women can provide the basis for engaging policy makers and service providers in the development of contextualized, evidence-based strategies to address overweight among women in Ghana and elsewhere with similar background.

## Materials and Methods

The current study was carried out in the four public and private health clinics providing maternal and child health services in the Dome Sub-district of the Ga East District of Ghana. Dome is mainly peri-urban with easy access to the business district of Ghana’s capital city, Accra. It also has relatively adequate access to public and private health services compared to other districts in Accra. Although the clinics were located in Dome, they also served nearby communities, including Ashongman, Aboso, and Taifa (20, 21). A cross-sectional design utilizing focus group discussions (FGDs) and in-depth interviews (IDI) were used for the study (refer to supplementary material). Qualitative data collection techniques were utilized in order to unearth the deep-seated beliefs and perceptions related to overweight and obesity.

Each of the clinics attend to an average of 50 postnatal women during the weekly child welfare clinic. From these clinics, women were invited to participate in the study if they were 18 years or older and agreed voluntarily to participate in the study. All the 42 respondents were urban-dwelling women who were accessing child care services from the clinics on the day of recruitment. Thirty-two of the women participated in four FGDs that involved an average of eight members per group. The FGD were all scheduled on a date other than the date of recruitment. The remaining 10 respondents were recruited to participate in the IDI. Women were approached if they appeared to be overweight to the researcher and discreetly invited to participate in the study, after having had explanation of the study procedures. Those women who self-identified as overweight, and agreed to participate by endorsing the informed consent form were recruited to participate in the IDI. Unlike the other women who participated, the overweight women were not brought together in a group discussion for two reasons: it was more convenient for them to be interviewed in their homes, and to avoid exposing them to stigmatization by other community members.

The FGDs were facilitated by a trained interviewer who was assisted by a note taker. On average, each FGD run for about 2 h. The facilitator led the group to explore their knowledge and perceptions on overweight, its determinants, beliefs about weight loss, and effect of weight loss on various dimensions of quality of life. The IDI that lasted for an average of one and a half hours each, explored the perceptions and experiences of the overweight women about their overweight status and how it has affected their quality of life. They were also asked questions on their experiences with weight loss. Both the FGD and IDI were conducted using an interview guide with open-ended questions. All the FGD and IDI were audio-taped, in addition to written notes. Evidence obtained from both the FGD and IDI were analyzed manually. The audio recordings were transcribed. The transcripts, together with the hand-written notes were manually read multiple times, by two independent investigators, coded and cross-referenced, and compared across the IDI and FGDs. Unique color coding and numbering was used to cluster similar themes and distinguish different themes. Frequency of the coded text was determined as indication of thematic unit significance. Their individual codes were then checked with each other for consistency and to arrive at consensus on the emergent themes. This allowed generalized perceptions and experiences that emerged from sorting the coded data to be pulled together in understanding the perceptions of the women using a combination of both systematic content analysis as well as narrative summary analysis (22, 23). The coding also allowed comparison of the themes across the IDI and FGD responses. Ethical approval for the study was obtained from the Ghana Health Service ethical review committee (Ethical Clearance ID: GHS-ERC:36/2/11). Written informed consent was provided by each participant after the study protocol had been carefully explained to them.

## Results

The study included adult women ranging from ages 18 to 47 years, a majority of whom were below 35 years (68%). Almost all the women (95%) were married. The findings of the study are organized according to the themes that emerged from the analysis relative to the study objectives (Table 1).

**Table 1:** **Comparison of perceptions and beliefs across focus group discussion and in-depth interview respondents**.

Key issues	Focus group discussion	In-depth interview
Perception of overweight	Stigmatizing language and behavior is used to characterize overweight and overweight persons when the person is considered extremely large or heavy	Stigmatization of overweight leads to poor self-image
		Stigma from overweight negatively affects social life, health status, and occupational functioning
	Some amount of weight gain is considered attractive and desirable and expected by society	Some amount of weight gain was expected by society and particularly by some spouses
	Desire for some amount of weight gain has not changed much over time	
	Weight gained deliberately using medication was considered undesirable	
Causes of overweight	Dietary habits (eating late dinners and uncontrolled appetite) and childbirth are considered main reasons for weight gain; also heredity and use of medication were given as reasons	Dietary habits (eating late dinners, consumption of energy-dense foods, and uncontrolled appetite) and childbirth are considered main reasons for weight gain; also, expectation by spouse to gain weight
Approaches and outcomes on weight gain		Reducing dietary intake quantity
		Exercise including jogging and participating in an exercise club
		Use of herbal medicines and Chinese tea

### Use of Stigmatizing Language in Reference to Overweight

A first step in all the study enquiries was to establish popular vocabulary for describing overweight. From the FGDs, the emerging theme was that overweight was typically described using stigmatizing language. It should be noted from this point onward, that unless otherwise stated, overweight is used to refer to all those with BMI perceived to be equal to or in excess of 25 kg/m^2^. In the FGDs, different words were used to describe overweight in the different Ghanaian languages. Most of the local language references to overweight can be translated to mean “big” which is typically a neutral word. However, when these words were applied to overweight individuals, the words were often used in a way that stigmatized people who are overweight. Examples identified include *okesie* (i.e., *big person in the Akan language*) and *maame agbo* (*big woman in the Ga language*). In addition to these, there were other words that were used in reference to overweight, which were metaphors describing something big. An example of this was *Cargo*. There were yet other terminologies that did not have obvious meaning but which were used in reference to a state of overweight and known across different languages. These included words, such as *Ngozie*, *Muchoomudoo*, and *Obolo*. Some of these words were derived from names used in reference to popular musicians, and television and cinema actors/actresses in Ghana (who are overweight) and, therefore, have assumed common use.

In the FGDs, some respondents indicated that, “*sometimes, the sight of an overweight person surprises you in a way that may lead you to say things that are not nice to them. This may be in the form of telling them that they are overweight or that they are gaining too much weight*.” In one of the groups, one respondent admitted that overweight persons suffer stigmatizing behavior and reminded the group that it was important to tell people about their overweight status in a careful way:

*Sometimes, some people do not know that they are fat. As time goes on, when the person is putting on more weight, they do not see it. So you as a friend, you have to call the person in a very polite way and tell the person that* (IDI #9).

In the IDI, the overweight women interviewed (8 out of 10) agreed that being called with such names made them feel stigmatized and uncomfortable. In addition, they also felt stigmatized by how other people looked at them and responded to their overweight status. As a result of being stigmatized, some of them indicated that they dreaded, “*going out*” and participating in social events. According to one of the respondents:

*Whenever I go out and there is laughter, I feel like am the cause of that laughter* (IDI #4).

The discussions altogether revealed exhibition of stigmatizing attitudes and behaviors toward persons who are overweight is common. Some respondents admitted that they have engaged in such stigmatization without realizing the adverse effect on those who are affected. The process of the discussing the issue and the potential harm such behavior was causing the overweight person allowed them to evaluate their attitudes.

### Perception of Overweight Status on Quality of Life

There were two key opposing general perceptions about persons who are overweight in the FGDs. It was believed that having some amount of weight was “*admirable*” and symbolizes a “*presentable*” body image. Having some weight was especially admired if it results in a “*proportional*” body frame. According to the respondents, some body weight was considered *proportional* if it was not limited to the upper parts of the body, but evenly distributed in every part of the body. Also, having some body weight for a person who is tall was perceived to be beautiful and to be admired. On the other hand, being short and overweight was not considered acceptable. Here is a quote from one of the FGD participants describing her view regarding gaining some weight:

*Yes, being big means that you are attractive. But there are different kinds of being big. There is the kind that makes you presentable. There is another kind of weight that makes you look disgusting and you feel uncomfortable* (FGD #1 participant).

All FGD participants were aware that some women deliberately gain weight by using “weight-gain” medication. This method of weight gain was perceived to be “*not proportional*” and, thus, not admirable. It emerged that the weight gained through the use of medication was often restricted to the buttocks, arms, and breasts. On the other hand, there was common belief among respondents that body weight that was proportional and, therefore, desirable was gained naturally without using medication. Although the overweight women in the IDI indicated their awareness of use of medication to gain weight, none of them admitted using medication to gain weight.

When asked whether Ghanaian women still cherished having some amount of weight, most group discussions arrived at the consensus that some amount of weight was still desired by many Ghanaian women. The suggestion that this perception was changing was rejected by the FGD participants. A recurring reason given for this opinion was that some women liked to be told that they had gained weight. The explanation given was that, having some amount of weight is perceived as a sign of “*enjoying good living*.”

*Yes, it still persists, even among our age group. When you reach a certain stage in life, you must look very attractive. When someone sees you, they say that you are looking very good* (FGD participant # 3).

While gaining some weight at any stage in life was considered to be desirable and was expected by society, this desire was even more socially expected during the first few months following key life stages, such as marriage and child birth. This perception had explanation in traditional norms among many ethnic groups in Ghana. Traditionally, it is expected that after becoming married, a husband will provide for the woman’s needs. A common indication of how well the husband provides for his spouse is how much weight the woman gains after marriage. This perception was frequently expressed in the FGDs and was also considered desirable among the IDI respondents: that is, gaining some amount of weight is evidence of a husband’s ability to adequately provide for the needs of his spouse. This expectation was considered so basic and essential that failure to demonstrate this weight gain can be recognized as a sign of financial weakness of the husband.

Both IDI and FGD respondents expressed the view that society expects women to gain weight following childbirth. Typically, a woman who has recently delivered is expected to have a certain plump body size that is needed to fit into the special clothing often worn during this period. According to one FGD respondent:

*But when you give birth, at least for the first three, four, five months, you need a little weight to look attractive* (FGD #1 participant).

An additional motivation for desiring some amount of body weight was the fear of being stigmatized by others as a woman who is slim. In extreme cases, slim women can become a target of name-calling in her house or community. In one FGD, it was mentioned that women who are slim were sometimes insulted as “*Nchanga*” (name of an extremely thin character in elementary school books), “*praye*” (meaning broom stick in the Akan language), or “*Chingilingi*” (no known meaning found), in the event of a domestic quarrel or in gossips. It was, therefore, suggested that the fear of being stigmatized or labeled as slim may explain why some women seek to gain weight.

However, it was also believed that persons who are overweight or heavy are slow and physically inactive, it was also thought that their excess body weight makes them tire easily. It was, commonly, indicated that persons who are overweight are handicapped. Another common perception was that overweight individuals always sit at one place, making it difficult for them to get up and perform usual activities.

Being overweight was thought to adversely affect a person’s social lifestyle. Particularly, references were made regarding the effect of overweight on marriage. There were accounts of situations where some overweight women had lost their husbands to other women who were not overweight. There was also the belief that overweight individuals are shy of people, although this sentiment was not shared by most of the women.

Being overweight was also linked with increased risk of disease. In particular, high blood pressure was mentioned as an outcome of being overweight. Two of the IDI respondents indicated that their overweight status was responsible for their having diabetes and hypertension.

### Beliefs about the Causes of Overweight

Both the IDI respondents and participants of the FGDs provided a variety of dietary- and non-dietary reasons why women become overweight. According to the focus group discussants, overweight among some women is an inherited condition. In making a case for heredity, it was argued by the discussion groups that some women were overweight because being overweight is a part of their families. Based on this belief, the weight gained by such women is considered “natural.” Some even believed that “*nothing can be done about it*” when overweight is inherited. Interestingly, none of the IDI participants shared this belief about overweight being heritable.

The perception that diet plays a role in being overweight was also widely held by both the IDI respondents and those in the FGDs. The association between diet and overweight was explained in terms of the types of foods that women ate, and also their eating habits and patterns. Particularly, eating late dinners and going to bed immediately, as well as having an uncontrolled appetite were thought to be important reasons why women became overweight. In addition, traditional diets consumed after child birth were especially singled out as a cause of overweight. In describing this particular reason, many of the women in the discussion groups expressed opinions suggesting at conflict in their perception. On the one hand, it was considered traditional in most Ghanaian communities to eat energy-dense (fatty) diets considered necessary for producing adequate breast milk for the newborn. Women, thus, felt obligated to eat these foods after delivery. On the other hand, they blamed such foods for making them gain more weight than they needed to.

*Our mothers told us that when you give birth, to a baby, for the first three months, you need to eat good food so that both you and the child will gain* (IDI #7).

Furthermore, it was reported that eating habits were more likely to result in weight gain if they were linked with physical inactivity. Indeed, it was commonly believed that going to bed immediately after eating promotes weight gain.

Among the IDI participants interviewed, only one indicated dietary habits as a reason for becoming overweight. All the remaining respondents linked their overweight status to their childbirth experience. Some of the respondents attributed their weight gain to the surgery (cesarean section) that was performed during childbirth. There was also the alternative view that weight gain resulted from the intravenous therapy that was given to them at the time they delivered.

Apart from the delivery-related reasons, some women in the FGDs identified the use of modern contraception as a reason for becoming overweight. There was one woman who added that drinking ice-cold water as a behavior could lead to weight gain.

In addition to these reasons that contribute to weight gain in a passive and unintentional manner, it was also revealed in the FGD that some women actively induced weight gain for a number of reasons. Chief among these was the need to satisfy the expectation of their spouse, regarding their appearance. There was a common belief that some husbands do not like “slim wives.” For a woman in such situation, if she failed to meet the expectation of the husband, she risked the husband cheating on her with a plump woman. One woman indicated that her husband has threatened her with divorce if she did not gain weight. Another woman in the IDI also said:

*my husband wants a fat person so he wants me to gain some more weight* (IDI #4).

It was believed that some women experience desire for weight gain for their own satisfaction. Among the women in the FGD, there was a perception that some amount of body weight was considered beautiful because it makes the woman “presentable.” There was also the perception that certain types of dresses were only beautiful on women if they had some amount of overweight. However, participants quickly added that the weight gained should not be too much.

### Experiences of Being Overweight

Most of the IDI respondents indicated that weight gain started only after delivery; some revealed that they used to be very slim until they had their first child. There was only one woman who indicated that she had been overweight for a long time. Also, only one of these women reported the reverse experience of weight loss during pregnancy that she re-gained after delivery.

Two of the IDI respondents reported that being overweight did not have any effect on their lives. One of these indicated that she felt overweight was a part of her. However, the rest of the IDI respondents expressed unhappy sentiments about how being overweight has affected their life, in four dimensions: health, work, social lifestyle, and self-image. Most of them linked their overweight status with having elevated blood pressure and diabetes during pregnancy. Concerning the relationship between overweight and work, they associated being overweight with becoming tired easily; they reported that being overweight slowed down their capacity to work, thus affecting their usual activities. Being overweight has also kept some of the women from having an active social lifestyle. Some indicated that they avoided going out to public places, whenever they could. Some were of the opinion that being overweight had made them anti-social. In addition, being overweight had created in them a poor self-image to a point where some of them now felt uncomfortable about how they looked. In addition, they had become very sensitive to what people say about them. According to one of the women:

*I don’t feel good about myself. I hate how I have become. All my friends were telling me I have grown too fat* (IDI #8).

### Weight Loss Experiences

All but two of the IDI respondents had tried shedding off some of their weight. Mostly, they were motivated to attempt weight loss when their husbands complained that they had gained too much weight. In addition, one person indicated that she had become slow in her work, and that was what made her decide to lose weight.

In order to lose weight, the women reported five approaches that they had attempted. Four of these were diet-related changes, including decreasing the total quantity of food consumed in a meal, reducing the frequency and amount of specific foods that are perceived as energy dense (*fufu, kenkey, and yam* were mentioned as examples), increasing the frequency and amount of fruit and vegetable consumption, and modifying the time of meals.

Most of the women who reported reducing total food consumed were essentially skipping breakfast, starving themselves until late afternoon before having any meal. However, those who practiced this approach reported that they had to eventually abandon this approach quickly. This was because they frequently experienced stomach ache due to long periods of starvation. Those who reported modifying their time of eating also frequently mentioned eating dinner earlier (before 04:00 p.m.). Only three women reported walking, jogging, or joining a “keep fit club” as a way of increasing their physical activity. However, all of them reported that their household chores did not permit them to continue these activities.

One woman mentioned wearing a corset but she also had to abandon its use after a short time because she was beginning to feel dizzy. Another woman reported using medication in her attempt to lose weight. Eventually, all the women admitted that their weight loss efforts failed, although three of the women indicated that they managed to slim down marginally. However, two of those who reported considerable weight loss indicated that they promptly gained back what they had lost. They also reported that others they knew had similar weight loss and regain experiences:

*My brother’s child has tried so hard, but has not lost weight. She loses it and gains it back. She controls herself but she is still big* (IDI # 6).

They identified barriers to losing weight as including: not knowing what to do, not having adequate support and help with their household chores, and losing motivation because the weight they lost was regained. One woman indicated that her work as a shop owner required her to sit for long hours. As a result, she rarely had opportunity to engage in physical activity.

The FGD participants recounted experiences of people they knew who had tried to lose weight using Chinese herbal medicines. According to them, these medicines were used to prepare tea for breakfast, to which nothing else is added. They also mentioned body creams that are used to rub the body as a way of stimulating weight loss. One woman testified that her sister reduced her weight by eating only fruits. The women were not in agreement, however, regarding the efficacy of these weight loss approaches. While some reported that these approaches mentioned were effective, others denied such claims.

*My sister has now reduced. I asked how she did it. She mentioned that she does not eat. She only takes in fruits* (FGD #1 participant).

## Discussion

In a culture in which, historically, more chronically undernourished adults have lived compared to those who were normal weight or overweight, studying perceptions about overweight is not a common enterprise. However, the increasing rates of overweight and obesity, particularly among women, makes such an exercise a critical necessity. The main finding of the current study is that women in the Ga East district recognize the multiple dimensions of the adverse effects of overweight as it affects social life, health, work, and self-image. Indeed, overweight individuals suffer name-calling and other embarrassing and stigmatizing behaviors as has been described elsewhere (11, 12). Nevertheless, strong positive perceptions of overweight originating from personal interest, societal expectation, and expectation of marital partners is also an important influence on women. While the desire to gain some weight is against the tide of popular culture, the expression of this desire provides useful evidence for public health intervention. Another common perception of interest was that being overweight is socially expected when individuals or families experience improved livelihoods. This finding has also been reported elsewhere (24). Altogether, the evidence suggests that the women have adopted a compromise view of overweight. This view says some amount of weight, but not too much of it, is socially admirable and considered presentable. About a decade ago, similar findings were reported among mostly young and lower class Senegalese women living in Dakar (25).

But the underlying conflict remains unresolved. An earlier study has reported that Ghanaian women perceived that their male partners wanted them to have a larger BMI, although both males and females desire a normal BMI (26). Interestingly, almost all the IDI respondents we interviewed wanted to reduce their weight in accordance with the findings of Duda et al. (27) And in most cases, the IDI respondents had, indeed, attempted to lose weight. Thus, there is an ongoing conflict regarding desire for a heavier body size versus the known adverse consequences of being overweight. Some of the conflict derives from cross-gender expectations that, as was demonstrated by Jumah and Duda (26), may not reflect what men and women really want for each other. Consistent with the findings in Ghana, evidence from elsewhere suggest that men and women view their partner’s weight status from different perspectives (28, 29). This finding suggests opportunity to explore weight management with a perspective on gender expectations of body weight.

Misperceptions regarding the reasons why women become overweight were commonly expressed in both the FGDs and by the IDI respondents. Such misperceptions were not surprising for a number of reasons. First, there is no structured framework to guide women and the entire Ghanaian public regarding prevention and control of excessive weight gain. Second, the absence of a control framework has allowed the proliferation of an unregulated industry that is marketing untested and unapproved remedies for weight reduction to Ghanaians. Along with these remedies, there was also widespread commercial promotion and dissemination, often via electronic media, of untested remedies for preventing and controlling overweight. Among the reasons indicated for gaining weight, dietary behavior was the most frequently mentioned. The women’s knowledge and perceptions regarding the role of diets was mostly consistent with reported evidence, although in some cases, there were misperceptions.

Our findings clearly show that many different remedies are being used by women, in their bid to reduce their weight. Often, these attempts were without successful outcomes. The lack of agreement among the study participants concerning the efficacy of these remedies suggest that, as expected, such remedies have poor success rates and, thus, do not deliver the promised outcomes regarding overweight control. Nevertheless, these remedies continue to be available through various sales outlets in Ghana, including pharmacies, chemical shops, supermarkets, corner stores, and other retail points. The proliferation of the weight reduction industry suggests the existence of an unmet need for policy and regulation to control the weight reduction industry. Such an intervention will provide guidance for the populace and also health care providers on evidence-based remedies for preventing and controlling overweight. Although a diet and physical activity guideline has been developed for Ghanaians, it fails to recommend guidelines on how to evaluate useful remedies for weight loss (30).

The findings of the current study are limited by inadequate characterization of the women who participated. In particular, information on the educational and socio-economic status of the participants, as well as data on objectively measured overweight status will have permitted better interpretation of the study. Furthermore, the current study did not differentiate between overweight and obesity. Considering that obesity is more strongly linked with both health and psychosocial factors, future studies will benefit from segregating evidence across overweight and obese subgroups. Nonetheless, the study was able to bring out key perceptions that can inform future studies and program planning and implementation.

## Conclusion

Although overweight is recognized as a threat to quality of life, some amount of body weight is admired and socially expected among some Ghanaian women as we have demonstrated in the Ga East district. Considering the rising levels of overweight in Ghana, there is need for policy and public health interventions to address overweight, particularly among women. In addition to interventions that address dietary energy intake, health education messages that help women to manage their perceptions of weight gain and that of their spouses will be an important aspect of a public health strategy to address overweight in Ghana and other similar settings. In the future, studies should further explore how family and marital relationships could be harnessed to promote appropriate communication and expectations about overweight.

### Implications for Research and Practice

Our findings on women’s beliefs about overweight suggest important social–cultural factors that must be considered when designing interventions to control overweight among Ghanaian women and other women living in similar social settings as the one described for this study. The misperceptions regarding the causes of weight gain are particularly important and should serve as useful feedback in behavior change communication, especially regarding diets and lifestyles. Together with other studies on body image among women and men in Ghana, it appears that social expectations of body image of women by their male partners play an important role in women’s perception of their body weight. Further research to clarify the basis for these expectations is warranted.

## Author Contributions

RA conceived the study, supervised study design and study tools, analyzed data, and prepared the manuscript.

## Conflict of Interest Statement

The author declares that the research was conducted in the absence of any commercial or financial relationships that could be construed as a potential conflict of interest. The reviewer (RP) and handling Editor declared their shared affiliation, and the handling Editor states that the process nevertheless met the standards of a fair and objective review.

## Supplementary Material

The Supplementary Material “Data collection tools” for this article can be found online at http://journal.frontiersin.org/article/10.3389/fnut.2016.00013
