# Supplementary material for: Perceptions and Experiences of Overweight among Women in the Ga East District, Ghana
Source: Front Nutr. 2016 Jun 2;3:13. doi: 10.3389/fnut.2016.00013 (PMC4889600; doi:10.3389/fnut.2016.00013)
Supplement: Supplementary file 1 [file Data_Sheet_1.DOCX]

**Data collection tools**

1. **FOCUS GROUP DISCUSSION GUIDE** (for woman attending CWC)
2. If someone is big or has ‘too much weight’, what are local words/phrases used to describe the person?
   1. Probe: What are the meanings/translations of those words?
3. When you look at someone, how can you tell that this person has excess weight?
4. When you see an overweight person, what comes to mind about the person?
   1. Probe: health status, lifestyle, work, relationship with other people, etc
5. What causes overweight?
   1. Probe: heredity, eating pattern, types of food, birth, medication, etc
6. There are stories about women who take medicines or do certain things that make them big, what do you know about this?
7. What do you know about experiences of others you know who are overweight or big?
8. There is a perception that Ghanaian women like to be gain weight or become big, for the purpose of fashion, social status, identifying with your friends, etc. What do you think about this?
9. What are the ways which you know that can make someone lose weight?
   1. Probe: food, Medicine, herbal/local remedies, exercise, Chinese medicine, etc
10. Do this weight reducing strategies work for everyone?
11. Do you know and would like to share the experiences of anyone who has tried to lose weight? How successful/unsuccessful were they?
12. **INTERVIEW GUIDE FOR OVERWEIGHT WOMEN** (those who consider themselves overweight and are considered by the investigator as overweight)
13. How long have you been heavy/big or had ‘much’ weight?
14. How do you feel about being big or having much weight?
    1. Probe: relating to your health, work, lifestyle, how others perceive your personality, etc?
15. What do you think made you heavy or have much weight?
16. If you have ever tried losing excess weight, what made you decide to do so?

Probe for other reasons

1. If you have ever tried losing weight, what did you do (or stop doing) to help you lose ‘excess’ weight?

Probe for other reasons

1. How successful were you in trying to lose ‘excess’ weight?
2. Which strategies for losing weight were useful and which were not? And why?
3. What are the experiences of others who have tried losing weight when they thought their weight was too much?
